# Calmodulin-like protein MdCML15 interacts with MdBT2 to modulate iron homeostasis in apple

**DOI:** 10.1093/hr/uhae081

**Published:** 2024-03-25

**Authors:** Xiao-Juan Liu, Xin Liu, Qiang Zhao, Yuan-Hua Dong, Qiangbo Liu, Yuan Xue, Yu-Xin Yao, Chun-Xiang You, Hui Kang, Xiao-Fei Wang

**Affiliations:** National Key Laboratory of Wheat Improvement, Apple Technology Innovation Center of Shandong Province, Shandong Green Fertilizer Technology Innovation Center, College of Horticulture Science and Engineering, Shandong Agricultural University, Tai-An, 271018, Shandong, China; State Key Laboratory of Tree Genetics and Breeding, Chinese Academy of Forestry, Beijing 100091, China; National Key Laboratory of Wheat Improvement, Apple Technology Innovation Center of Shandong Province, Shandong Green Fertilizer Technology Innovation Center, College of Horticulture Science and Engineering, Shandong Agricultural University, Tai-An, 271018, Shandong, China; Institute of Forestry and Pomology, Academy of Agriculture and Forestry Sciences, Beijing 100093, China; National Key Laboratory of Wheat Improvement, Apple Technology Innovation Center of Shandong Province, Shandong Green Fertilizer Technology Innovation Center, College of Horticulture Science and Engineering, Shandong Agricultural University, Tai-An, 271018, Shandong, China; College of Horticulture, Qingdao Agricultural University, Qingdao 266109, China; National Key Laboratory of Wheat Improvement, Apple Technology Innovation Center of Shandong Province, Shandong Green Fertilizer Technology Innovation Center, College of Horticulture Science and Engineering, Shandong Agricultural University, Tai-An, 271018, Shandong, China; National Key Laboratory of Wheat Improvement, College of Life Sciences, Shandong Agricultural University, Tai-An, 271018, China; State Key Laboratory of Tree Genetics and Breeding, Chinese Academy of Forestry, Beijing 100091, China; National Key Laboratory of Wheat Improvement, Apple Technology Innovation Center of Shandong Province, Shandong Green Fertilizer Technology Innovation Center, College of Horticulture Science and Engineering, Shandong Agricultural University, Tai-An, 271018, Shandong, China; National Key Laboratory of Wheat Improvement, Apple Technology Innovation Center of Shandong Province, Shandong Green Fertilizer Technology Innovation Center, College of Horticulture Science and Engineering, Shandong Agricultural University, Tai-An, 271018, Shandong, China; National Key Laboratory of Wheat Improvement, Apple Technology Innovation Center of Shandong Province, Shandong Green Fertilizer Technology Innovation Center, College of Horticulture Science and Engineering, Shandong Agricultural University, Tai-An, 271018, Shandong, China; National Key Laboratory of Wheat Improvement, Apple Technology Innovation Center of Shandong Province, Shandong Green Fertilizer Technology Innovation Center, College of Horticulture Science and Engineering, Shandong Agricultural University, Tai-An, 271018, Shandong, China

## Abstract

BTB and TAZ domain proteins (BTs) function as specialized adaptors facilitating substrate recognition of the CUL3–RING ubiquitin ligase (CRL3) complex that targets proteins for ubiquitination in reaction to diverse pressures. Nonetheless, knowledge of the molecular mechanisms by which the apple scaffold protein MdBT2 responds to external and internal signals is limited. Here we demonstrate that a putative Ca ^2+^ sensor, calmodulin-like 15 (MdCML15), acts as an upstream regulator of MdBT2 to negatively modulate its functions in plasma membrane H^+^-ATPase regulation and iron deficiency tolerance. MdCML15 was identified to be substantially linked to MdBT2, and to result in the ubiquitination and degradation of the MdBT2 target protein MdbHLH104. Consequently, MdCML15 repressed the MdbHLH104 target, *MdAHA8*’s expression, reducing levels of a specific membrane H^+^-ATPase. Finally, the phenotype of transgenic apple plantlets and calli demonstrated that MdCML15 modulates membrane H^+^-ATPase-produced rhizosphere pH lowering alongside iron homeostasis through an MdCML15–MdBT2–MdbHLH104–MdAHA8 pathway. Our results provide new insights into the relationship between Ca^2+^ signaling and iron homeostasis.

## Introduction

Iron (Fe), an indispensable micronutrient necessary for photosynthetic, respiratory, and hormone synthesis processes and for nitrogen fixation and hemoglobin synthesis [1]. Although Fe is abundant in the soil, it usually exists in the form of insoluble ferric hydroxides, especially in calcareous soil, resulting in the low Fe availability. This restricted Fe availability seriously harms crop yields and human health [2]. To adapt to Fe limitation, plants have been selected to generate two primary strategies for the acquisition of soil-localized Fe. The strategy I response is a reduction-based strategy in non-graminaceous plants, in which Fe^3+^ is solubilized via H^+^-ATPase, which acidifies the rhizosphere, and the iron is then reduced to Fe^2+^ through the action of the ferric chelate reductase known as FERRIC REDUCTION OXIDASE2 (FRO2). Subsequently, Fe^2+^ is introduced into roots via the metal transporter IRON REGULATED TRANSPORTER1 (IRT1) [3, 4]. The strategy II response is a chelation-based strategy in graminaceous plants, in which Fe^3+^ is chelated by phytosiderophores (mugineic acids, MAs) to form Fe^3+^–MA complexes. The complexes are then carried into the root through the transporters known as YELLOW STRIPE1/YELLOW STRIPE1-LIKE (YS1/YSL) [5, 6].

Transcriptional regulation plays an important role in the Fe-deficient responses, and a number of transcription factors (TFs) that modulate a set of Fe-deficient response genes have been identified, particularly the basic helix–loop–helix (bHLH) TFs [7, 8]. In *Arabidopsis*, the bHLH TF IIIa subgroup FER-LIKE IRON DEFICIENCY-INDUCED TRANSCRIPTION FACTOR (FIT) is able to form a heterodimer with diverse Ib bHLHs (including bHLH38, bHLH39, bHLH100, and bHLH101), to co-regulate the expression of *IRT1* and *FRO2* to control Fe uptake and responses [9, 10]. POPEYE (PYE/bHLH47), a IVb subgroup bHLH TF, is a major player in maintaining Fe homeostasis through the inhibition of genes linked to Fe transport as well as homeostasis, such as *NICOTIANAMINE SYNTHASE 4* (*NAS4*) and *FRO3* [11]. Four bHLH TFs of the IVc subgroup, including IAA-LEUCINE RESISTANT3 (ILR3, also called bHLH105), bHLH34, bHLH104, and bHLH115, as well as the IVb subgroup bHLH121 are characterized as directly activating the transcription of *PYE* and *bHLH38/39/100/101* involved in regulating the Fe deficiency response [12–17]. In apple (*Malus domestica*) and citrus, MdbHLH104 and MYB308 regulate rhizosphere acidification and Fe absorption by mediating the expression of plasma membrane *Autoinhibited H^+^-ATPase* (*AHA*) genes [18, 19].

In addition to transcriptional regulation, post-translational modification acting upstream of these TFs in response to Fe deficiency has also been extensively investigated. For instance, the RING E3 ubiquitin ligase named BRUTUS (BTS) and its paralogs, including BTS LIKE1 (BTSL1) and BTSL2 in *Arabidopsis*, as well as its orthologs HEMERYTHRIN MOTIF-CONTAINING RING AND ZINC-FINGER PROTEIN1 (OsHRZ1) in rice are found to ubiquitinate and degrade bHLH TFs (ILR3 and bHLH115, FIT, and POSITIVE REGULATOR OF IRON HOMEOSTASIS1 (OsPRI1), respectively), thereby negatively regulating Fe deficiency responses [11, 15, 20–23]. In apple, MdbHLH104 was recruited by the BTB/TAZ protein MdBT2 for degradation and sumoylated by the SUMO E3 ligase MdSIZ1, to co-control its stability to regulate PM H^+^-ATPase-caused rhizosphere pH lowering, as well as Fe absorption in response to Fe deficiency stress [24, 25]. Interestingly, recent studies found that in *Malus xiaojinensis*, an Fe-efficient apple rootstock, ROS-induced MxMPK6 could phosphorylate MxbHLH104 to promote the Fe deficiency tolerance [26, 27].

BTs belong to the BTB/TAZ subfamily of BTB/POZ proteins, which usually function as substrate-specific adaptors to bind Cullin 3 (CUL3), forming CUL3–RING ubiquitin ligase (CRL3) complexes, and target proteins for ubiquitination [28, 29]. In *Arabidopsis*, there are five members of the BTB/TAZ subfamily, from BT1 to BT5 [30]. Each member contains a conserved N-terminal BTB domain for interacting with the CUL3 complex, as well as a central TAZ domain for recruiting target proteins, and a C-terminal calmodulin-binding domain (CaMBD), which may be responsible for binding CaMs in a Ca^2+^-dependent manner [31, 32]. BT2 is able to react to several hormones and stresses, including the introduction of auxin, abscisic acid (ABA), nutrients, and circadian stress [33, 34]. Ca^2+^ is thought to regulate *35S* enhancer-mediated transcription through the CaMBD domain of BT2, suggesting that Ca^2+^ may be sensed via CaMs in response to diverse signals [35]. When BT2 acts as a downstream target of TELOMERASE ACTIVATOR 1 (TAC1) to regulate telomerase activity, it is activated by increased Ca^2+^ concentrations [36]. BT2 also interacts with Ca^2+^-dependent protein kinases (CPKs) such as CPK4 and CPK11 [37], but the effects of these interactions have not been identified. These findings suggest an association between BT2 and Ca^2+^ signaling; however, the regulatory mechanisms involved are still poorly understood.

In apple, MdBT2 is a key component involved in various activities, such as Fe homeostasis, nitrogen utilization, anthocyanin biosynthesis, malate accumulation, and abiotic stress [38]. Here we searched for the MdBT2 interacting protein in apple through yeast two-hybrid (Y2H) screening, and identified a CaM-like (CML) protein, MdCML15. CaM/CML families contain EF-hand domains that trap Ca^2+^ and thus transduce calcium signaling, and are widely involved in regulating plant growth and development and coping with environmental stress [39, 40]. Recent studies have shown that MdCaM4 mediates phosphorylation of MdCYTOKININ RESPONSE FACTOR4 (MdCRF4), leading to ubiquitination degradation of MdCRF4, ultimately inhibiting ethylene biosynthesis during apple fruit ripening [41]. In the Fe-efficient rootstock *Malus xiaojinensis*, the MxMPK4-1 cascade is activated by Ca^2+^/MxCaM7 signaling, which phosphorylates MxIQM3, thereby promoting the separation of MxIQM3 from MxAHA2 and enhancing H^+^ secretion under Fe deficiency stress [42]. In this study, we found that MdCML15 negatively regulated Fe deficiency tolerance by promoting MdBT2-mediated MdbHLH104 degradation. Our results provide evidence for the relationship between Ca^2+^/CaM, BT2, and Fe homeostasis.

## Results

### CaM-like protein MdCML15 is identified as a putative MdBT2 interaction partner

**Figure 1:**
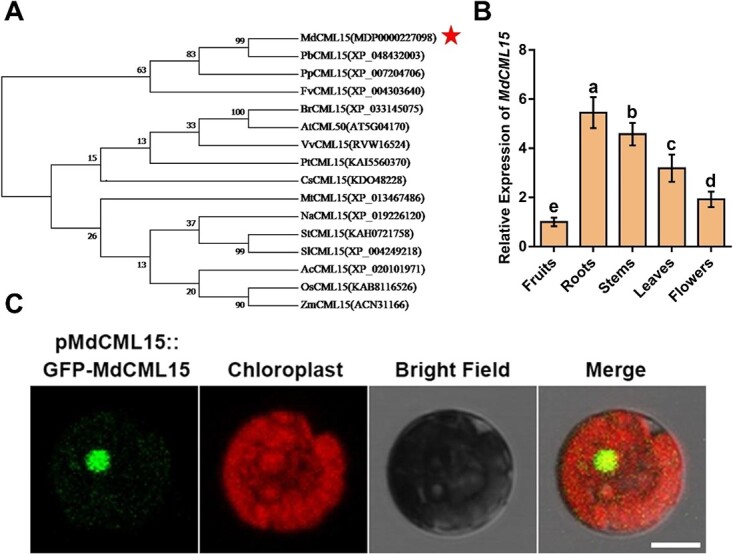
Expression pattern and subcellular localization of MdCML15. **A** Phylogenetic tree analysis of MdCML15 and 15 other plants’ CML15 protein sequences obtained from the NCBI database. MdCML15 is denoted by the red asterisk. PbCML15, *Pyrus bretschneideri*; PpCML15, *Prunus persica*; FvCML15, *Fragaria vesca*; CsCML15, *Citrus* spp.; MtCML15, *Medicago truncatula*; NaCML15, *Nicotiana attenuata*; SlCML15, *Solanum lycopersicum*; StCML15, *Solanum tuberosum*; AcCML15, *Ananas comosus*; VvCML15, *Vitis vinifera*; AtCML15, *Arabidopsis thaliana*; BrCML15, *Brassica rapa*; PtCML15, *Populus trichocarpa*; OsCML15, *Oryza sativa*; ZmCML15, *Zea mays*. **B** Expression analysis of *MdCML15* in apple roots, stems, leaves, flowers, and fruits. Data are represented as relative to fruits. Results shown are mean ± standard deviation based on three independent biological replicates. Different letters represent significant differences (Tukey’s test, *P* < 0.05). **C** Subcellular localization of MdCML15 in GL-3 apple leaf protoplasts. Scale bar = 10 μm.

To determine whether there is an association between BT2 and Ca^2+^ signaling, we screened the apple cDNA library with Y2H using MdBT2 as bait. A cDNA fragment of *MDP0000227098* encoding part of the CaM-like protein was screened out. According to the chromosomal localization nomenclature of MdCMLs [43], the gene was named *MdCML15*. To know the evolutionary relationships of CML15s in plants, we made a phylogenetic tree and found that MdCML15 is most closely related to CML15s from other Rosaceae plants, followed by PbCML15, PpCML15, and FvCIP8 (Fig. 1A). Reverse transcription–quantitative PCR (RT–qPCR) examination demonstrated that *MdCML15* was expressed throughout roots, stems, leaves, flowers, and fruits, with the highest transcript level in roots (Fig. 1B). Further, GUS staining of *pMdCML15::GUS* transgenic *Arabidopsis thaliana* showed different expression levels of *MdCML15* in various tissues (Supplementary Data Fig. S1). We also detected the subcellular localization of MdCML15, and found that the green fluorescence signal of *pMdCML15::GFP-MdCML15* mainly accumulated in the nucleus of apple leaf protoplasts (Fig. 1C).

### MdCML15 interacts with MdBT2

**Figure 2:**
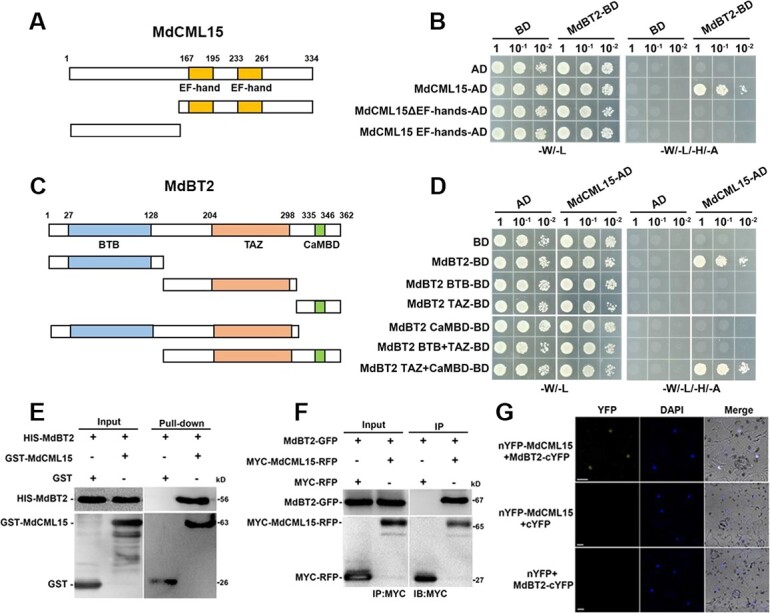
MdCML15 interacts with MdBT2. **A** Diagram of EF-hand domains in MdCML15 protein. **B** Y2H analysis between MdCML15 segments and full-length MdBT2. **C** Diagram of BTB, TAZ, and CaMBD domains in MdBT2 protein. **D** Y2H analysis between MdBT2 segments and full-length MdCML15. **E** Pull-down assay of the interaction between MdCML15 and MdBT2. HIS-MdBT2 was incubated with GST-MdCML15 or GST. GST was used as the negative control. **F** Co-IP assay of the interaction between MdCML15 and MdBT2. Total protein was extracted from *35S::MdBT2-GFP + 35S::MYC-MdCML15-RFP* and *35S::MdBT2-GFP + 35S::MYC-RFP* transgenic calli. **G** BiFC assay of the interaction between MdCML15 and MdBT2 in epidermal cells of tobacco leaves. nYFP–MdCML15 + cYFP and nYFP + MdBT2–cYFP served as negative controls. Nuclei are marked with DAPI. Scale bars = 10 μm.

MdCML15, a protein that interacts with MdBT2, was selected through Y2H screening. Subsequently, various experiments were performed to verify the contact taking place between MdCML15 and MdBT2. First, we performed a Y2H assay to detect the MdCML15–MdBT2 interaction and its key domains. MdCML15 contains two EF-hand domains (Fig. 2A). Y2H results showed that full-length MdCML15 interacts with MdBT2, but the EF-hand domain alone cannot (Fig. 2B). MdBT2 consists of the N-terminal BTB domain, the middle TAZ domain, and the C-terminal CaMBD domain (Fig. 2C). MdBT2 fragments containing the TAZ and CaMBD domains interact with MdCML15, while MdBT2 fragments lacking the CaMBD domain or the TAZ domain do not (Fig. 2D), suggesting that these two domains were essential for the MdCML15–MdBT2 interaction. In addition to MdBT2, MdCML15 also interacts with MdBT1; however, it does not network with any of MdBT3.1, MdBT3.2, or MdBT4 (Supplementary Data Fig. S2). Moreover, MdBT2 interacts specifically with MdCML subfamily VIII members such as MdCML15, MdCML16, MdCML58 and MdCML60; it does not interact with other MdCMLs (Supplementary Data Fig. S3).

Next, we conducted a pull-down experiment to determine whether MdCML15 physically interacts with MdBT2 *in vitro*. Prokaryotic-produced GST proteins or GST-MdCML15 recombinant proteins were incubated with HIS–MdBT2 proteins. HIS–MdBT2 was obtained using GST–MdCML15 but not when using GST alone (Fig. 2E), suggesting that MdCML15 is linked to MdBT2 *in vitro*. For the Co-IP assay, the immunoprecipitated proteins were analyzed with anti-MYC and anti-GFP antibodies. MdBT2–GFP was detected in the *35S::MdBT2-GFP + 35S::MYC-MdCML15-RFP* calli but not in the *35S::MdBT2-GFP + 35S::MYC-RFP* calli (Fig. 2F), indicating that MdCML15 interacts with MdBT2 *in vivo*. Then a bimolecular fluorescence complementation (BiFC) assay was performed to confirm the MdCML15–MdBT2 interaction *in vivo*. As a result, nYFP–MdCML15 + MdBT2–cYFP produced strong yellow fluorescence signal in the nucleus, but no signal was observed in the negative control nYFP–MdCML15 + cYFP or nYFP + MdBT2–cYFP (Fig. 2G), indicating that MdCML15–MdBT2 interaction occurs in the nucleus.

### MdCML15 negatively modulates PM H^+^-ATPase-linked rhizosphere pH lowering and Fe insufficiency tolerance

MdBT2 is involved in regulating PM H^+^-ATPase activity and Fe deficiency response through its interaction with MdbHLH104, which directly activates the transcription of *MdAHA8* [18, 24]. Since MdCML15 interacts with MdBT2, we investigated the role of MdCML15 in response to Fe deficiency. First, we tested whether MdCML15 responds to different iron treatments at transcript and protein levels. RT–qPCR analysis showed that the transcription level of *MdCML15* was significantly downregulated after −Fe + Frz treatment (Supplementary Data Fig. S4A). Moreover, we used a cell-free protein degradation assay to detect the abundance of MdCML15 protein. Western blot results showed that, compared with +Fe treatment, −Fe + Frz treatment accelerated the degradation of HIS–MdCML15 protein (Supplementary Data Fig. S4B). In addition, the degradation of MdCML15 was significantly inhibited by the proteasome inhibitor MG132 (Supplementary Data Fig. S4B). Thus, these findings suggest that Fe deficiency inhibits MdCML15 expression at the transcriptional and post-translational levels.

**Figure 3:**
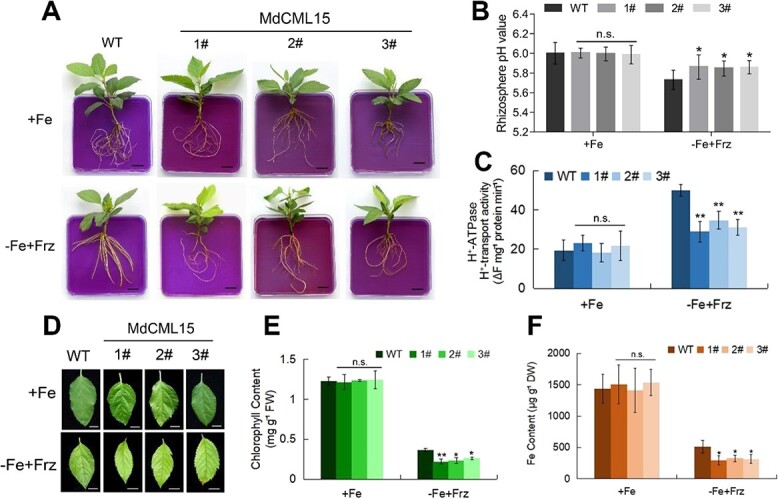
Transgenic apple seedlings overexpressing *MdCML15* are more sensitive to Fe deficiency. **A** Rhizosphere acidification of 30-day-old WT and *35S::MdCML15* transgenic lines given +Fe (Fe-sufficient) or −Fe + Frz (Fe-deficient, without Fe and supplemented with 100 μM ferrozine) treatment for 10 days. The plants were then transferred to a flat containing bromocresol purple for 1 day; the yellow color represents rhizosphere acidification. Scale bars = 1 cm. **B** Root rhizosphere pH values of WT and *35S::MdCML15* transgenic plants under +Fe or −Fe + Frz treatment for 2 days. **C** The PM H^+^-ATPase activity of 30-day-old WT and *35S::MdCML15* transgenic lines treated with +Fe or −Fe + Frz for 10 days. **D** Young leaves of 30-day-old WT and *35S::MdCML15* transgenic lines after exposure to +Fe or −Fe + Frz medium for 3 weeks. Scale bars = 5 mm. **E** Total chlorophyll content of the young leaves in (**D**). FW, fresh weight. **F** Fe content of the young leaves in (**D**). DW, dry weight. In (**B**), (**C**), (**E**) and (**F**), error bars indicate standard deviation of three independent biological replicates. n.s., *P* > 0.05, ^*^P < 0.05, ^**^P < 0.01.

Subsequently, we generated transgenic plants with overexpression of *MdCML15* (*35S::MdCML15*). The expression of *MdCML15* in *35S::MdCML15-1#/2#/3#* was 7–10 times that of the wild type (WT; Supplementary Data Fig. S5). We treated the *35S::MdCML15-1#/2#/3#* transgenic plants with Fe sufficiency or Fe deficiency for 30 days. The pH indicator bromocresol purple visualizes PM H^+^-ATPase-mediated rhizosphere acidification. In contrast to the WT, the *35S::MdCML15-1#/2#/3#* lines exhibited reduced rhizosphere acidification when exposed to Fe-deficient conditions, as evidenced by the less intense yellow hue of the medium surrounding the roots (Fig. 3A). Additionally, all three *35S::MdCML15* lines demonstrated increased rhizosphere pH and decreased activity of the PM H^+^-ATPase compared with the WT when subjected to Fe deficiency (Fig. 3B and C). We also observed that the young leaves of the *35S::MdCML15-1#/2#/3#* lines exhibited more severe chlorosis and lower chlorophyll and Fe contents than those of WT when exposed to Fe deficiency (Fig. 3D–F). Nonetheless, no significant variance was observed between the three *35S::MdCML15* transgenic lines and WT (Fig. 3). These results suggested that MdCML15 negatively regulates Fe deficiency tolerance.

**Figure 4:**
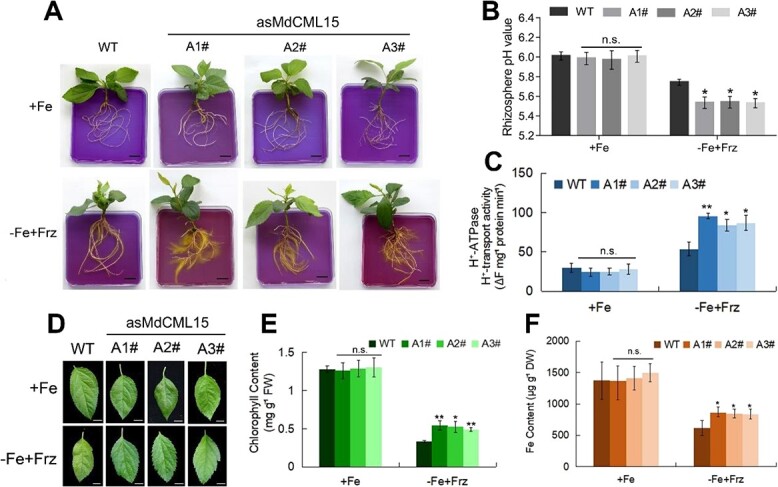
Transgenic apple seedlings antisense-expressing *MdCML15* are tolerant to Fe deficiency. **A** Rhizosphere acidification of 30-day-old WT and *35S::asMdCML15* transgenic lines treated with +Fe or −Fe + Frz for 10 days. The plants were then transferred to a flat containing bromocresol purple for 1 day; the yellow color represents rhizosphere acidification. Scale bars = 1 cm. **B** Root rhizosphere pH values of WT and *35S::asMdCML15* transgenic plants under +Fe or −Fe + Frz treatment for 2 days. **C** PM H^+^-ATPase activity of 30-day-old WT and *35S::asMdCML15* transgenic lines treated with +Fe or −Fe + Frz for 10 days. **D** Young leaves of 30-day-old WT and *35S::asMdCML15* transgenic lines after exposure to +Fe or −Fe + Frz medium for 3 weeks. Scale bars = 5 mm. **E** Total chlorophyll content of the young leaves in (**D**). FW, fresh weight. **F** Fe content of the young leaves in (**D**). DW, dry weight. In (**B**), (**C**), (**E**) and (**F**), error bars indicate standard deviation of three independent biological replicates. n.s., *P* > 0.05, ^*^P < 0.05, ^**^P < 0.01.

We also obtained transgenic plants with antisense expression of *MdCML15* (*35S::asMdCML15*). The expression of *MdCML15* in *35S::asMdCML15-A1#/A2#/A3#* was 40% that of WT (Supplementary Data Fig. S5). Moreover, the transcripts of *MdCML16*, *MdCML60*, and *MdCML58* homologous genes of *MdCML15* did not change significantly in *35S::asMdCML15-A1#/A2#/A3#*, indicating that the three *35S::asMdCML15* transgenic lines were specific to *MdCML15 *(Supplementary Data Fig. S6). The results showed that compared with WT, the *35S::asMdCML15-A1#/A2#/A3#* lines displayed more rhizosphere acidification, lower rhizosphere pH and higher PM H^+^-ATPase activity under Fe deficiency conditions (Fig. 4A–C). Moreover, the three *35S::asMdCML15* lines had less chlorosis and higher chlorophyll and Fe contents than WT when subjected to Fe-deficient conditions (Fig. 4D–F). Nevertheless, no significant variability was observed between the three *35S::asMdCML15* lines and WT under Fe-sufficient conditions (Fig. 4). These results suggested that MdCML15 plays negative roles in Fe deficiency tolerance.

In addition, *35S::MdCML15* and *35S::asMdCML15* transgenic calli were used to further examine the function of MdCML15 (Supplementary Data Fig. S7A). Compared with WT, *35S::MdCML15* overexpression calli exhibited less rhizosphere acidification, lower PM H^+^-ATPase activity, and lower Fe content under Fe deficiency conditions, while *35S::asMdCML15* suppression calli exhibited the opposite phenotype (Supplementary Data Fig. S7B–E), indicating that MdCML15 negatively modulates the PM H^+^-ATPase-caused rhizosphere pH lowering as well as Fe insufficiency tolerance.

### MdCML15 is involved in MdBT2-mediated degradation of MdbHLH104

**Figure 5:**
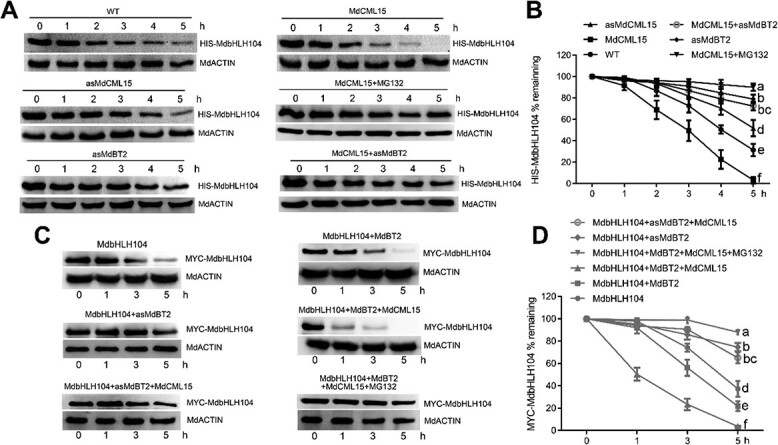
MdBT2 is required for MdCML15-induced MdbHLH104 degradation. **A** MdBT2 was required for MdCML15-induced MdbHLH104 degradation *in vitro*. Total proteins from WT, *35S::MdCML15* (MdCML15), *35S::asMdCML15* (asMdCML15), *35S::asMdBT2* (asMdBT2), and *35S::MYC-MdCML15 + 35S::asMdBT2* (MdCML15 + asMdBT2) transgenic calli were incubated with HIS−MdbHLH104 protein. The mixture was collected at the indicated times and detected with anti-HIS antibody. **B** The abundance of HIS−MdbHLH104 protein in (**A**) was quantified with Bio-Rad Quantity One software. **C** MdBT2 was required for MdCML15-induced MdbHLH104 degradation *in vivo*. *MdCML15* was transiently overexpressed in *35S::MYC-MdbHLH104* + *35S::MdBT2* (MdbHLH104 + MdBT2) and *35S::MYC-MdbHLH104* + *35S::asMdBT2* (MdbHLH104 + asMdBT2) transgenic calli. Calli were collected at the indicated times and detected with anti-MYC antibody. **D** Abundance of MYC−MdbHLH104 protein in (**C**) was quantified with Bio-Rad Quantity One software. MdACTIN was used as a loading control. In (**B**) and (**D**) different letters represent significant differences (Tukey’s test, *P* < 0.05) and error bars indicate standard deviation of three biological replicates.

In our previous studies, MdBT2 negatively modulated MdbHLH104 protein abundance by mediating its ubiquitination and degradation [24]. We therefore examined whether MdCML15 affects the abundance of MdbHLH104 protein *in vitro* and *in vivo*. First, a cell-free degradation assay was carried out. Recombinant HIS−MdbHLH104 protein was incubated with extract from WT, *35S::MdCML15* and *35S::asMdCML15* calli. The findings demonstrated that the HIS−MdbHLH104 protein was broken down more rapidly in *35S::MdCML15* extract, while this process took a longer time in *35S::asMdCML15* extract compared with WT (Fig. 5A and B), indicating that MdCML15 promotes the degradation of MdbHLH104 protein. Further, the degradation of HIS−MdbHLH104 in *35S::MdCML15* extract was inhibited by the proteasome inhibitor MG132 (Fig. 5A and B). Moreover, compared with WT, HIS−MdbHLH104 degraded slower in *35S::asMdBT2* extract (Fig. 5A and B). When *MdCML15* was overexpressed in *35S::asMdBT2* calli, the degradation of HIS−MdbHLH104 was slower than that in *35S::MdCML15* calli, similar to that in *35S::asMdBT2* calli (Fig. 5A and B; Supplementary Data Fig. S8A), suggesting that MdCML15-induced MdbHLH104 degradation requires MdBT2 *in vitro*.

To assess the impact of MdCML15 on the levels of MdbHLH104 protein *in vivo*, we employed a viral vector, pIR, to induce transient overexpression of *MdCML15* in calli containing *35S::MYC-MdbHLH104 + 35S::MdBT2-GFP* and *35S::MYC-MdbHLH104 + 35S::asMdBT2* (Supplementary Data Fig. S8B). The quantification of MdbHLH104 protein abundance was conducted using an anti-MYC antibody. The findings revealed that MYC–MdbHLH104 protein degradation occurred at a swifter pace in *35S::MYC-MdbHLH104 + 35S::MdBT2-GFP* calli compared with *35S::MYC-MdbHLH104* calli. Conversely, the degradation rate was slower in *35S::MYC-MdbHLH104 + 35S::asMdBT2* calli (Fig. 5C and D), signifying that MdBT2 facilitates the *in vivo* degradation of MdbHLH104 protein. Moreover, MYC−MdbHLH104 degradation was accelerated in *35S::MYC-MdbHLH104 + 35S::MdBT2-GFP + pIR-MdCML15* calli when compared with *35S::MYC-MdbHLH104* + *35S::MdBT2-GFP + pIR* calli (Fig. 5C and D), suggesting that MdCML15 enhances the MdBT2-mediated degradation of MdbHLH104 protein. Notably, the degradation of MYC−MdbHLH104 in *35S::MYC-MdbHLH104 + 35S::MdBT2-GFP + pIR-MdCML15* calli was nearly completely inhibited by MG132 (Fig. 5C and D). Additionally, no differences were observed in the degradation of MYC−MdbHLH104 between *35S::MYC-MdbHLH104 + 35S::asMdBT2 + pIR-MdCML15* and *35S::MYC-MdbHLH104 + 35S::asMdBT2 + pIR* calli (Fig. 5C and D), indicating that MdBT2 is required for MdCML15-mediated MdbHLH104 degradation *in vivo*.

### MdCML15 promotes MdBT2-mediated MdbHLH104 ubiquitination

**Figure 6:**
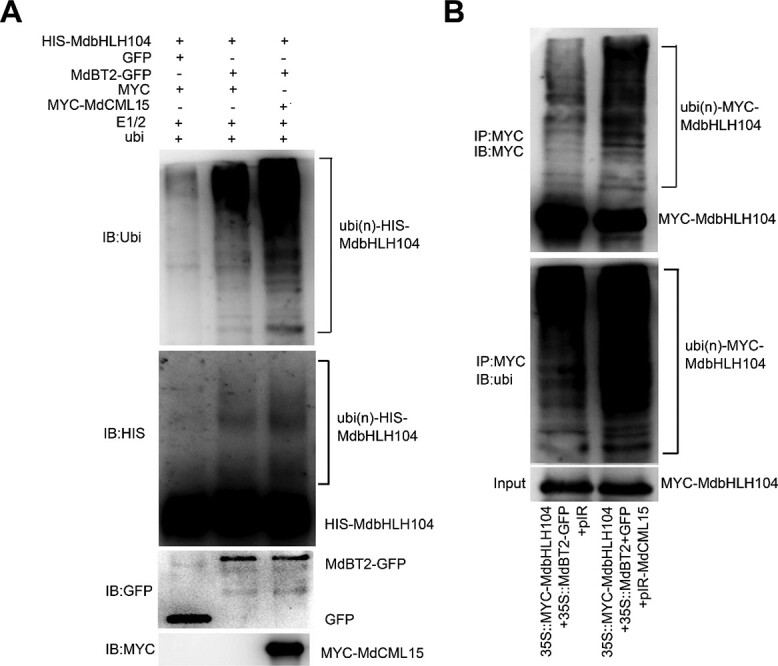
MdCML15 promotes MdBT2-mediated MdbHLH104 ubiquitination. **A** MdCML15 promoted MdBT2-mediated MdbHLH104 ubiquitination *in vitro*. HIS−MdbHLH104 protein in a mixture prior to reaction was detected with anti-HIS antibody and used as input. The indicated reaction products were detected with anti-HIS and anti-ubiquitin antibodies, respectively. Ubi(n)-HIS−MdbHLH104 refers to polyubiquitinated HIS−MdbHLH104 protein. **B** MdCML15 promoted MdBT2-mediated MdbHLH104 ubiquitination *in vivo*. The viral vector pIR-MdCML15 was transformed into *35S::MYC-MdbHLH104* + *35S::MdBT2-GFP* transgenic calli. The MYC−MdbHLH104 protein was immunoprecipitated with anti-MYC antibody and analyzed with anti-MYC and anti-ubiquitin antibodies. The empty vector pIR served as a negative control. Input refers to the MYC−MdbHLH104 protein prior to reaction detection with anti-MYC antibody.

To examine whether MdCML15 affects MdBT2-mediated ubiquitination of MdbHLH104 protein, we carried out ubiquitination assays *in vitro* and *in vivo*. In a semi-*in vitro* ubiquitination assay, the purified HIS−MdbHLH104 protein was used as substrate. MdBT2−GFP and MYC−MdCML15 proteins were immunoprecipitated using anti-GFP and anti-MYC antibodies, targeting the proteins in *35S::MdBT2-GFP* and *35S::MYC-MdCML15* calli, respectively (Fig. 6A). Subsequently, the ubiquitination of the MdbHLH104 protein was assessed using anti-ubiquitin and anti-HIS antibodies. Western blotting results showed that HIS−MdbHLH104 was ubiquitinated when MdBT2−GFP was present, and strengthened by MYC−MdCML15 (Fig. 6A), indicating that MdCML15 promotes MdBT2-mediated ubiquitination of MdbHLH104 *in vitro*.

To further detect the effect of MdCML15 on MdBT2-mediated MdbHLH104 ubiquitination *in vivo*, the viral vector *pIR-MdCML15* was rapidly transformed into *35S::MYC-MdbHLH104* + *35S::MdBT2-GFP* calli (Supplementary Data Fig. S8B). Subsequently, the MYC−MdbHLH104 protein was subjected to immunoprecipitation using an anti-MYC antibody, with samples collected from both *35S::MYC-MdbHLH104 + 35S::MdBT2-GFP + pIR* and *35S::MYC-MdbHLH104 + 35S::MdBT2-GFP + pIR-MdCML15* calli. The ubiquitination of the MdbHLH104 protein was assessed utilizing both anti-MYC and anti-ubiquitin antibodies. The results clearly demonstrated that the ubiquitination level of MYC−MdbHLH104 protein in *35S::MYC-MdbHLH104 + 35S::MdBT2-GFP + pIR-MdCML15* calli was significantly higher compared with that in *35S::MYC-MdbHLH104 + 35S::MdBT2-GFP + pIR* calli (Fig. 6B). This observation underscores the role of MdCML15 in enhancing the ubiquitination of MdbHLH104 protein mediated by MdBT2 *in vivo*.

### MdCML15 negatively regulates PM H^+^-ATPase activity and Fe deficiency tolerance in an MdBT2-dependent manner

**Figure 7:**
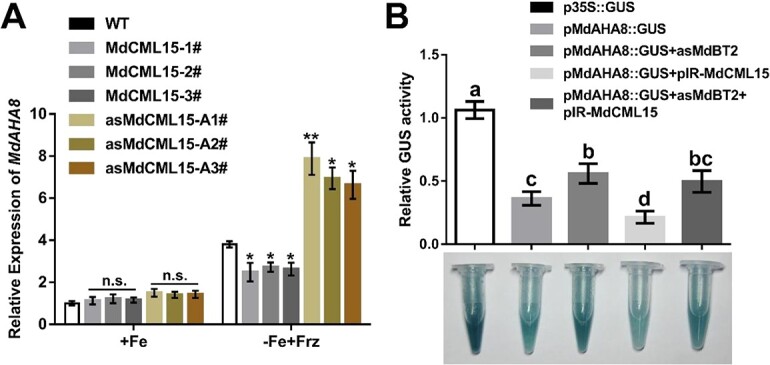
MdCML15-inhibited *MdAHA8* expression depends on MdBT2. **A** RT–qPCR analysis of *MdAHA8* in the roots of WT, *35S::MdCML15*, and *35S::asMdCML15* transgenic plants. Error bars indicate standard deviation of three biological replicates. n.s., *P* > 0.05, ^*^P < 0.05, ^**^P < 0.01. **B** GUS staining and GUS activity analysis of *MdAHA8* promoter using *pMdAHA8::GUS* in calli. The viral vector pIR-MdCML15 was transformed into *pMdAHA8::GUS* and *pMdAHA8::GUS + 35S::asMdBT2* transgenic calli. GUS activity was measured using a 4-methylumbelliferyl-d-glucuronide method. The GUS activity of *p35S::GUS* was set to 1. Different letters represent significant differences (Tukey’s test, *P* < 0.05) and error bars indicate standard deviation of three biological replicates.

MdbHLH104 directly activates *MdAHA8* expression by binding to its promoter [18]. Since MdCML15 negatively regulates MdbHLH104 protein stability, we then investigated whether MdCML15 affects the expression of *MdAHA8*. The results showed that, compared with WT, the transcript level of *MdAHA8* was lower in the roots of *35S::MdCML15-1#/2#/3#* overexpression plants but higher in *35S::asMdCML15-A1#/A2#/A3#* suppression plants in response to Fe deprivation (Fig. 7A). Moreover, we also determined the expression levels of *MdIRT1* and *MdFRO2*, which play key roles in Fe^3+^ reduction and Fe^2+^ absorption. RT–qPCR results showed that after iron deficiency treatment MdCML15 could partially inhibit the expression of *MdIRT1* and *MdFRO2*, but the degree of inhibition was smaller than that of *MdAHA8* (Supplementary Data Fig. S9). To explore the role of MdBT2 in MdCML15-inhibited *MdAHA8* expression, *pIR-MdCML15* was transiently transformed into *pMdAHA8::GUS* and *pMdAHA8::GUS* + *35S::asMdBT2* calli under Fe deficiency conditions (Supplementary Data Fig. S10A). GUS staining and activity analysis results showed that the *MdAHA8* promoter activity was higher in *pMdAHA8::GUS* + *35S::asMdBT2* calli than in *pMdAHA8::GUS* calli (Supplementary Data Fig. 7B). Meanwhile, overexpression of *MdCML15* inhibited the *MdAHA8* promoter activity in *pMdAHA8::GUS + pIR-MdCML15* calli, while inhibition of *MdBT2* in *pMdAHA8::GUS + 35S::asMdBT2 + pIR-MdCML15* calli significantly eliminated this effect (Fig. 7B). These results indicated that MdCML15 negatively regulates *MdAHA8* expression in an MdBT2-dependent manner.

**Figure 8:**
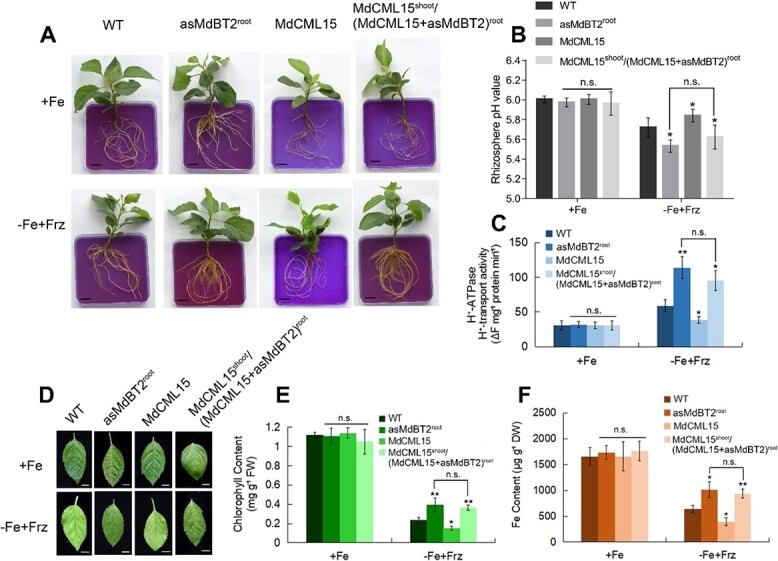
MdCML15-modulated PM H^+^-ATPase activity and Fe homeostasis requires MdBT2. **A** Rhizosphere acidification of 1-month-old plants under +Fe or −Fe + Frz conditions for 10 days. Acidification was visualized with bromocresol purple. Scale bars = 1 cm. **B** Root rhizosphere pH values of plants in (**A**) under +Fe or −Fe + Frz treatments for 2 days. **(C)** PM H^+^-ATPase activity of plants in (**A**). **D** Young leaves of 1-month-old plants exposed to +Fe or −Fe + Frz conditions for 20 days. Scale bars = 5 mm. **E** Total chlorophyll content of the young leaves in (**D**). FW, fresh weight. **F** Fe content of the young leaves in (**D**). DW, dry weight. In (**B**), (**C**), (**E**) and (**F**), error bars indicate standard deviation of three biological replicates. n.s., *P* > 0.05, ^*^P < 0.05, ^**^P < 0.01.

To investigate the function of MdBT2 in MdCML15 inhibition of the action of PM H^+^-ATPase and Fe insufficiency tolerance, *MdBT2* was repressed within the roots of WT and *35S::MdCML15* transgenic apple plantlets by an *Agrobacterium rhizogenes*-mediated method to generate *35S::asMdBT2*^root^ and *35S::MdCML15*^shoot^/(*35S::MdCML15* + *35S::asMdBT2*)^root^ plantlets (Supplementary Data Fig. S10B). In response to Fe deprivation, the *35S::asMdBT2*^root^ plantlets exhibited lower rhizosphere pH, higher PM H^+^-ATPase activity, more obvious rhizosphere acidification, higher Fe content, and greener young leaves compared with WT (Fig. 8). Meanwhile, the *35S::MdCML15* plantlets showed the opposite phenotype, such as lower PM H^+^-ATPase activity, weaker rhizosphere acidification, lower Fe content, and more leaf chlorosis (Figs 3 and 8). However, *MdBT2* suppression in *35S::MdCML15*^shoot^/(*35S::MdCML15* + *35S::asMdBT2*)^root^ transgenic roots exhibited the phenotype of *35S::asMdBT2*^root^ plantlets, largely abolishing the effects of MdCML15 (Fig. 8). These results illustrated that MdBT2 is necessary for MdCML15-mediated responses to Fe deficiency.

Finally, transgenic calli *35S::asMdBT2*, *35S::MdCML15*, and *35S::MdCML15 + 35S::asMdBT2* were used to further examine the role of MdBT2 in MdCML15 function (Supplementary Data Fig. S11). *MdBT2* suppression partially abolished MdCML15 effects related to PM H^+^-ATPase-governed pH lowering and Fe content in *35S::MdCML15* + *35S::asMdBT2* calli in response to Fe-deficient conditions (Supplementary Data Fig. S11), suggesting that MdBT2 is required for the function of MdCML15.

## Discussion

Ca^2+^ represents a ubiquitous second messenger with roles in various stages of growth and development in plants. Ca^2+^ signaling is recognized and decoded by Ca^2+^ sensors and then transmitted to downstream effectors to induce a series of physiological and biochemical processes [39]. Three primary categories of Ca^2+^ sensors exist, namely (i) calmodulins (CaMs) and calmodulin-like proteins (CMLs), (ii) calcineurin B-like proteins (CBLs), and (iii) calcium-dependent protein kinases (CPKs) [40]. In recent years, there have been a few reports on the mechanism of Ca^2+^ signaling regulating Fe homeostasis. In *Arabidopsis*, CPK21 and CPK23 phosphorylate and activate the iron transporter IRT1 to promote Fe absorption [44]. The Ser/Thr protein kinase CBL-INTERACTING PROTEIN KINASE11 (CIPK11), which is activated by Ca^2+^-CBL1/9, targets and phosphorylates FIT to adjust Fe acquisition [45]. In *Malus xiaojinensis*, an Fe-efficient apple rootstock, MxCaM7, decodes Ca^2+^ signaling to interact with MxIQM3, and simultaneously Ca^2+^ signaling activates the MxMPK4-1 cascade to phosphorylate MxIQM3 at the Ser^393^ site, thus causing MxIQM3 to dissociate from MxAHA2 and enhancing H^+^ secretion under Fe deficiency stress [42]. Interestingly, our results revealed that MdCML15 negatively regulates PM H^+^-ATPase-governed rhizosphere pH lowering and Fe insufficiency tolerance by interacting with MdBT2 and promoting MdBT2-mediated degradation of MdbHLH104 (Figs 2–6). Thus, plants maintain Fe homeostasis through different Ca^2+^ sensor-mediated regulatory pathways.

The CML proteins typically contain one to six EF-hand domains that bind Ca^2+^, and participate in diverse life processes, including polar cell growth, flowering time, and tolerance to drought, cold, and nutrient stresses [46, 47]. For example, CML23 and CML24 regulate flowering process, and CML38 regulates root growth in *A. thaliana* [48, 49], while MtCML10 and MtCML42 modulate cold tolerance in *Medicago truncatula* [50, 51]. Here we found that MdCML15 maintains Fe homeostasis in apple (Fig. 3), indicating that the functions of CMLs are extensive and diverse in plants. MdCML15 belongs to the VII subfamily of MdCMLs, and other members of the MdCML VIII subfamily, including MdCML16, MdCML58, and MdCML60, also interact with MdBT2 (Supplementary Data Fig. S3), indicating that MdCML15 may exhibit functional redundancy with MdCML16, MdCML58, or MdCML60. However, *35S::asMdCML15* suppression plants showed stronger resistance to Fe deficiency than WT (Fig. 4), suggesting that MdCML15 plays a major role, or that MdCML16, MdCML58, or MdCML60 may regulate other MdBT2-mediated activities.

As a key component of substrate recognition in the CRL3 ubiquitin complex, BTB/TAZ proteins regulate multiple plant growth and development and stress response processes [29]. There are five MdBT members in the apple genome, all of which contain the CaMBD domain [52]. In addition to MdBT2, MdCML15 also interacts with MdBT1 (Supplementary Data Fig. S2). Among them, MdBT2 has been the most widely studied and plays a central role in mediating various environmental and hormonal signals to regulate apple growth and development [38]. For example, MdBT2 regulates Fe homeostasis, anthocyanin biosynthesis, malate accumulation, leaf senescence, plant growth, and nitrogen usage by interacting with MdbHLH104, MdMYB1, MdCIbHLH1, MdMYB73, MdZAT10, MdRGL3a, and MdMYB88/124 proteins, and promoting their ubiquitination and degradation [24, 52–57]. However, the function of the CaMBD domain was not mentioned. In this study, we found that MdCML15 interacts with the CaMBD domain of MdBT2, albeit with the help of the TAZ domain (Fig. 2D), which may be due to the fact that the CaMBD domain is too small in the spatial conformation, containing 12 amino acids, to support the interaction with the entire protein of MdCML15, and therefore requires the assistance of the neighboring domain TAZ. Meanwhile, MdCML15 negatively regulates Fe deficiency tolerance by promoting MdBT2-mediated degradation of MdbHLH104 protein (Figs 2–6). CMLs are Ca^2+^ sensors and are capable of transducing calcium signaling [39, 40]. Our study not only clarified the role of the CaMBD domain, but also established the relationship between calcium signaling and MdBT2-mediated Fe homeostasis, and even other functions.

**Figure 9:**
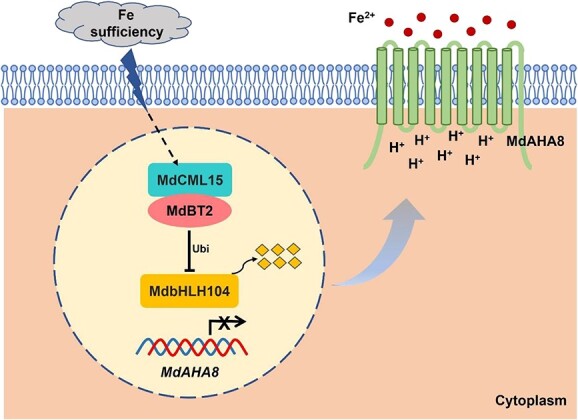
A model of Fe homeostasis regulation by MdCML15–MdBT2–MdbHLH104–MdAHA8 in apple trees. When apple roots are exposed to Fe-sufficient conditions, MdCML15 is induced to interact with MdBT2 in the nucleus to accelerate MdBT2-mediated ubiquitination and degradation of MdbHLH104, relieve transcriptional activation of *MdAHA8*, reduce H^+^ efflux, and thereby inhibit Fe absorption and prevent Fe toxicity.

Apple stands as a pivotal economic fruit crop worldwide and ranks as one of the most susceptible fruit trees to Fe deficiency. Consequently, investigating the regulatory mechanisms governing Fe homeostasis in apple holds paramount significance. It was found that MdbHLH104 significantly enhanced Fe uptake by transcriptionally activating *MdAHA8* expression and promoting H^+^ efflux [18]. Subsequently, three forms of protein modification for MdbHLH104 were uncovered. MdSIZ1 and MxMPK6 induced by Fe deficiency can sumoylate and phosphorylate MdbHLH104, respectively, and enhance the transcriptional activity of MdbHLH104, thereby promoting Fe absorption and coping with Fe deficiency stress [25, 26]. However, the ubiquitination modification of MdbHLH104 by MdBT2 occurs under Fe-sufficient conditions, where MdBT2 was induced to ubiquitinate and degrade MdbHLH104, preventing excessive Fe from being toxic to plants [24]. Our results suggest that MdCML15 negatively modulates Fe deficiency tolerance by interacting with MdBT2 and promoting MdBT2-mediated degradation of MdbHLH104 (Figs 2–6). Based on the above, we propose a simple model (Fig. 9). When apple roots are exposed to Fe-sufficient conditions, MdCML15 is induced to interact with MdBT2 in the nucleus to accelerate MdBT2-mediated ubiquitination and degradation of MdbHLH104, relieve transcriptional activation of *MdAHA8*, reduce H^+^ efflux, and thereby inhibit Fe absorption and avoid Fe toxicity. Our study not only enriched the regulatory network of Fe absorption, but also provided evidence for the link between calcium signaling and Fe homeostasis.

## Materials and methods

### Plant resources and growth environments

Apple ‘Gala’ tissue cultures were employed as the control or WT plants in this study. These apple plants were cultivated at a temperature of 25°C under long-day conditions (16 h of light and 8 h of darkness) for 1 month. For subculturing, the growth medium employed was Murashige and Skoog (MS) supplemented with 1 mg l^−1^ of 6-benzylaminopurine (6-BA), 0.2 mg l^−1^ of naphthylacetic acid (NAA), and 0.5 mg l^−1^ of gibberellic acid (GA). Once rooted, a 1/2 MS medium supplemented with 1 mg l^−1^ of indole-3-acetic acid (IAA) was used. ‘Orin’ apple calli were cultured on MS medium supplemented with 1.5 mg l^−1^ of 2,4-dichlorophenoxyacetic acid (2,4-D) and 0.4 mg l^−1^ of 6-BA under darkness at 25°C and were subcultured at 3-week intervals.

For Fe starvation treatments, apple plants were transferred after rooting to Fe-sufficient (+Fe) liquid 1/2 MS medium or Fe-deficient (−Fe + Frz) liquid medium (1/2 MS without Fe supplemented with 100 μM ferrozine) for 10 or 20 days. The 3-week-old calli were transferred to +Fe medium (normal calli medium) or −Fe + Frz medium (normal callus medium without Fe supplemented with 100 μM ferrozine) for 15 days.

### Vector construction and genetic transformation

To construct *MdCML15* and *MdBT2* overexpression vectors, the *MdCML15* coding sequence (CDS) was linked with PXSN-MYC and PXSN vectors to form *35S::MYC-MdCML15* and *35S::MdCML15*, and the *MdBT2* CDS was linked with PRI–GFP vector to construct *35S::MdBT2-GFP*. To construct the *MdCML15* and *MdBT2* silencing vectors, 200–300 bp specific antisense fragments of *MdCML15* and *MdBT2* were linked with PXSN or PRI to generate *35S::asMdCML15* and *35S::asMdBT2*, respectively. The promoter fragment (2000 bp) of *MdCML15* was connected with 1300-GUS to generate *pMdCML15::GUS*. Subsequently, the genetic transformation of apple tissue cultures and calli was performed using *Agrobacterium tumefaciens* containing the recombinant plasmids [58]. Transgenic *35S::MYC-MdbHLH104* and *35S::MYC-MdbHLH104* + *35S::MdBT2-GFP* calli were described in Zhao *et al*. [24].

For transient transformation, *MdCML15* CDS was connected with the virus vector pIR [59]. IL-60-BS was used as auxiliary constructor to transform apple callus for 1 week together with pIR-MdCML15 or pIR.

For genetic analysis, the 200–300 bp specific antisense fragment of *MdBT2* was cloned into pK7GWIWG2 to generate *35S::asMdBT2*. The *35S::asMdBT2* vector was introduced into *A. rhizogenes* MSU440 and then genetically transformed into transgenic apple plantlets using an MSU440-mediated method [60]. The infected roots were cultivated using MS medium supplemented with 300 mg l^−1^ cephalosporin for 20 days. Then the transgenic roots were assessed by fluorescence screening, and rooting seedlings were chosen for phenotypic analysis.

### Quantitative real-time PCR

Total RNA was extracted using the RNA Plant Plus Reagent (Tiangen, China). Subsequently, 1 μg of RNA was used to synthesize cDNA with the PrimeScript™ first-strand cDNA synthesis kit (Takara, Japan). A qRT–PCR assay was conducted using SYBR Green (Takara, Japan) and an ABI7500 system. The specific primers are detailed in Supplementary Data Table S1.

### Phylogenetic tree analysis

The protein sequences from other species were blasted using MdCML15 as bait in the NCBI database (https://www.ncbi.nlm.nih.gov/). The AtCML50 (AT5G04170) protein sequence was downloaded from the *Arabidopsis* website (http://www.arabidopsis.org/). All protein sequences were multiple sequence aligned and used to construct a phylogenetic tree via the MEGA7 software.

### Yeast two-hybrid screening and assays

Y2H screening and Y2H assays were carried out following the manufacturer’s guidelines (Clontech). The complete cDNA sequence of *MdBT2*, along with different segments of the gene, was cloned into the bait vector pGBT9. Additionally, the full-length cDNAs of *MdBT1*, *MdBT3.1*, *MdBT3.2*, and *MdBT4* were also inserted into the bait vector. The complete cDNA alongside the domain deletion of *MdCML15*, as well as the full-length cDNAs of *MdCaM4*, *MdCML12*, *MdCML19*, *MdCML24*, *MdCML30*, *MdCML58*, *MdCML16*, *MdCML60*, *MdCML65*, *MdCML71*, and *MdCML76*, were inserted into the pGAD424 vector.

The Y2H screening experiment was carried out using MdBT2 as bait, and single colonies on −Trp−Leu−His−Ade (−W−L−H−A) medium were sequenced. Potential interacting proteins were identified by blasting the sequencing results. For Y2H assays, the resultant constructs were transformed into yeast, and yeast were grown on −W−L medium and screened using a −W−L−H−A medium.

### Pull-down assay

To obtain recombinant protein, the *MdCML15* cDNA was introduced into the pET32a vector, while the *MdBT2* cDNA was integrated into the pGEX-4 T-1 vector. These constructed plasmids, along with the pGEX-4 T-1 vector itself, were introduced into *Escherichia coli* BL21 (DE3) for the expression of HIS–MdCML15, GST–MdBT2, and GST proteins, respectively. In the pull-down assay, 6 μg of HIS–MdCML15 was incubated with 10 μg of either GST–MdBT2 or GST. The pull-down procedure was performed using the Pierce GST Spin Purification Kit (Thermo).

### Co-immunoprecipitation assay

Co-IP method was according to Kang *et al*. [61]. *35S::MYC-RF*P and *35S::MYC-MdCML15-RFP* were transiently transformed into *35S::MdBT2-GFP* transgenic calli and cultured for 3 days for the Co-IP experiment. The calli were pretreated with MG132 for 1 h. Total proteins directly detected by anti-MYC and anti-GFP antibodies were used as input. The proteins were immunoprecipitated with an anti-MYC antibody, and then the immunoprecipitated proteins were detected with anti-GFP and anti-MYC antibodies.

### Bimolecular fluorescence complementation assay

Full-length CDS sequences of *MdCML15* and *MdBT2* were inserted into the pSITE–nYFP and pSITE–cYFP vectors to create the nYFP–MdCML15 and MdBT2–cYFP fusions, respectively. The plasmids were then transformed into *A. tumefaciens* GV3101, and the indicated combinations were transiently co-transformed into *Nicotiana benthamiana* leaves [61]. The YFP fluorescence was observed after 2-day infiltration with a Zeiss LSM880 high-resolution laser confocal microscope.

### Subcellular localization in apple leaf protoplasts

The transient transformation of apple leaf protoplasts was performed by a PEG-mediated method. *pMdCML15::GFP-MdCML15* plasmids were transformed into protoplasts of 3-week-old apple tissue culture seedlings. After 16 h of light culture, the GFP fluorescence was observed with a Zeiss LSM880 high-resolution laser confocal microscope.

### Rhizosphere pH measurements

The method of rhizosphere pH measurement was according to Sun *et al*. [62]. Apple seedlings were transferred after rooting to −Fe or −Fe + Frz liquid medium (pH = 6.0) for 2 days. The pH meter was slowly moved closer to the rhizosphere and the pH value was recorded when the value was stable. At least three plants were selected for rhizosphere pH determination.

### GUS assay

For GUS histochemical staining, the *pMdAHA8::GUS* associated calli were submerged in staining buffer (0.075 M sodium phosphate buffer at a pH of 7.0, containing 0.05 mM ferricyanide, 0.05 mM ferrocyanide, 10 mM EDTA, 0.1% Triton X-100, 20% methanol, and 1 mM X-Gal) at a temperature of 37°C for 6 h. The GUS activity of calli was assessed as outlined by Liu *et al*. [58].

### *In vitro* and *in vivo* degradation examination

Twenty-day-old transgenic calli grown in normal medium were subjected to extraction using a degradation buffer composed of 25 mM Tris–HCl (pH 7.5), 5 mM DTT, 4 mM PMSF, 10 mM ATP, 10 mM MgCl_2,_ and 10 mM NaCl [63]. These samples were employed for cell-free protein degradation assays. Equal amounts of total proteins were co-incubated with prokaryotic-induced protein at 22°C, and samples were subsequently collected in loading buffer at specified time points.

To conduct degradation assays *in vivo,* total proteins were extracted from 20-day-old calli cultivated in normal medium and treated with cycloheximide, a protein synthesis inhibitor. Equal protein amounts were incubated at 22°C at the indicated time intervals. For the 26S proteasome inhibition assay, the calli were pretreated with 50 μM MG132, a proteasome inhibitor, for 1 h. Quantification of target proteins was carried out with the corresponding antibodies.

### Ubiquitination assays

In order to conduct ubiquitination assays *in vivo, 35S::MdBT2-GFP* transgenic calli cultured under normal medium were subjected to a 1-h treatment with MG132. The reaction system (30 μl) comprised 200 ng HIS-MdbHLH104, 400 ng MdBT2–GFP or GFP, 500 ng MYC–MdCML15 or MYC, 25 ng UBE1 (E1; Boston Biochem), 50 ng E2 UbcH5b (E2; Boston Biochem), and 1 μg ubiquitin in a ubiquitination buffer (composed of 2 mM DTT, 50 mM Tris–HCl (pH 7.5), 5 mM MgCl_2_, and 2 mM ATP). This mixture was incubated for 16 h at 30°C. The samples taken before the reaction were assessed using an anti-HIS antibody (input), while the reaction products were subsequently analyzed using anti-HIS and anti-ubiquitin antibodies.

For the ubiquitination assay *in vivo*, *35S::MYC-MdbHLH104* + *35S::MdBT2-GFP* or *35S::MYC-MdbHLH104* + *35S::asMdBT2* calli, which transiently co-expressed pIR or pIR-MdCML15 for 1 week under normal callus medium, were initially pretreated with 50 μM MG132 for 1 h. They were then incubated for 6 h. The MYC–MdbHLH104 proteins were immunoprecipitated using an anti-MYC antibody. Detection of the immunoprecipitated proteins was carried out using anti-MYC and anti-ubiquitin antibodies.

### PM H^+^-ATPase activity and rhizosphere acidification assays

The PM H^+^-ATPase activity assay was conducted as previously outlined [25]. Calli that were treated with +Fe or −Fe + Frz for 15 days, as well as apple plantlets subjected to with +Fe or −Fe + Frz for 10 days, were utilized to isolate PM. PM proteins were combined with the enzyme reaction buffer (containing 25 mM 1,3-bis [tris (hydroxylmethyl) methylamino] propane-HEPES (pH 6.5), 3 mM MgSO_4_, 100 mM KCl, 10 μM quinacrine, and 250 mM mannitol) for 5 min in darkness. The reaction was initiated by adding 3 mM ATP, and fluorescence was measured at 430 nm excitation and 500 nm emission. At the conclusion of the reaction, 10 μM m-chlorophenylhydrazone (CCCP) was introduced. Finally, GUS activity was computed as the change in fluorescence per minute per unit mass of PM protein.

For the rhizosphere acidification assay, calli treated with +Fe or −Fe + Frz for 15 days, and apple plantlets subjected to +Fe or -Fe + Frz for 10 days, were transferred to plates containing 0.006% bromocresol purple, 0.2 mM CaSO_4_, and 1% agar adjusted to pH 6.5 for 1 day.

### Accession numbers

Sequence data utilized in this article can be accessed from the Genome Database for Rosaceae (GDR; https://www.rosaceae.org) data libraries under the accession numbers MdBT2 (MDP0000643281), MdCML15 (MDP0000227098), MdbHLH104 (MDP0000825749), MdAHA8 (MDP0000181085), MdBT1 (MDP0000151000), MdBT4 (MDP0000215415), MdCaM4 (MDP0000865414), MdCML12 (MDP0000380330), MdCML19 (MDP0000859814), MdCML24 (MDP0000817646), MdCML30 (MDP0000164511), MdCML58 (MDP0000664492), MdCML16 (MDP0000120294), MdCML60 (MDP0000178485), MdCML65 (MDP0000602146), MdCML71 (MDP0000176317), MdBT3.1 (MDP0000296225), MdBT3.2 (MDP0000187156), and MdCML76 (MDP0000583167).

## Acknowledgements

We would like to sincerely thank our team leader, Dr Yu-Jin Hao. He will be remembered for his great achievement and for his support and assistance in this work. This work was supported by grants from National Key Research and Development Program (2022YFD1201701), the National Natural Science Foundation of China (32001336 and 32272683), the China Agriculture Research System of MOF and MARA (CARS-27), and the Natural Science Foundation of Shandong Province (ZR2022QC093).

## Author contributions

C.X.Y and X.F.W. planned and designed the research. X.J.L., H.K., X.L., Q.Z., and Y.H.D. performed experiments and analyzed the data. Q.L., Y.X., and Y.X.Y. provided technical assistance. X.J.L., H.K., and X.F.W. wrote the article.

## Data availability

The data underlying this article are available in the manuscript and in its online supplementary material.

## Conflict of interest

The authors declare that no competing interests exist.

## Supplementary data

Supplementary data are available at *Horticulture Research* online.

## Supplementary Material

Web_Material_uhae081
