# Supplementary material for: Calmodulin-like protein MdCML15 interacts with MdBT2 to modulate iron homeostasis in apple
Source: Hortic Res. 2024 Mar 25;11(5):uhae081. doi: 10.1093/hr/uhae081 (PMC11101318; doi:10.1093/hr/uhae081)
Supplement: Web_Material_uhae081 [file web_material_uhae081.zip › Supplementary data.pdf]

Supplementary data

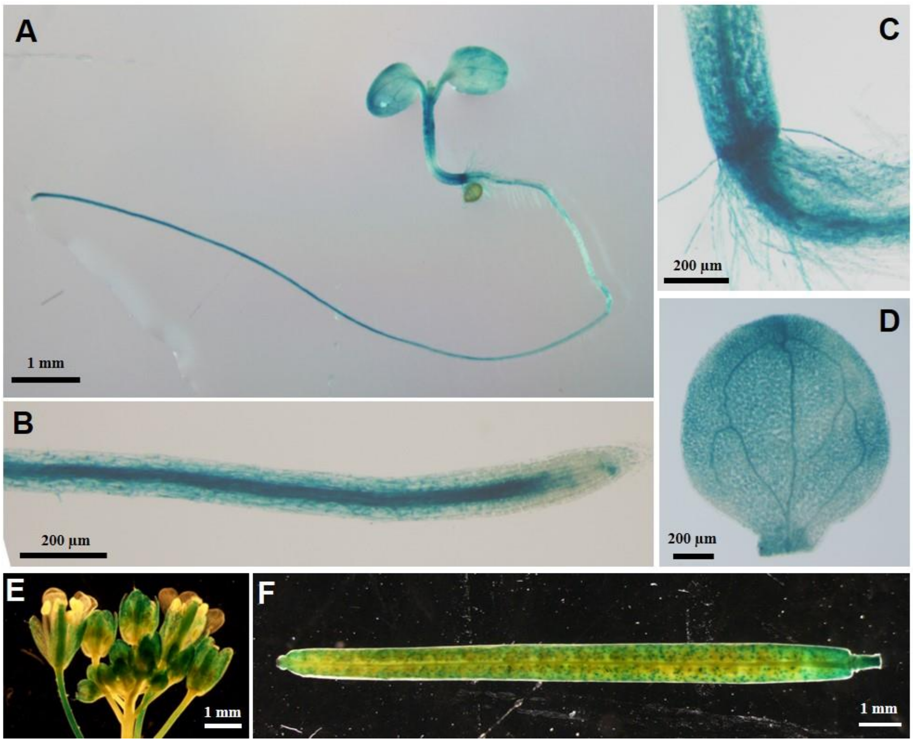

Figure S1 GUS staining of *pMdCML15::GUS* transgenic *Arabidopsis*.

(A) Four-day old seedlings; **(B)** Roots; **(C)** Hypocotyls; **(D)** Leaves; **(E)** Inflorescences; **(F)** Siliques.

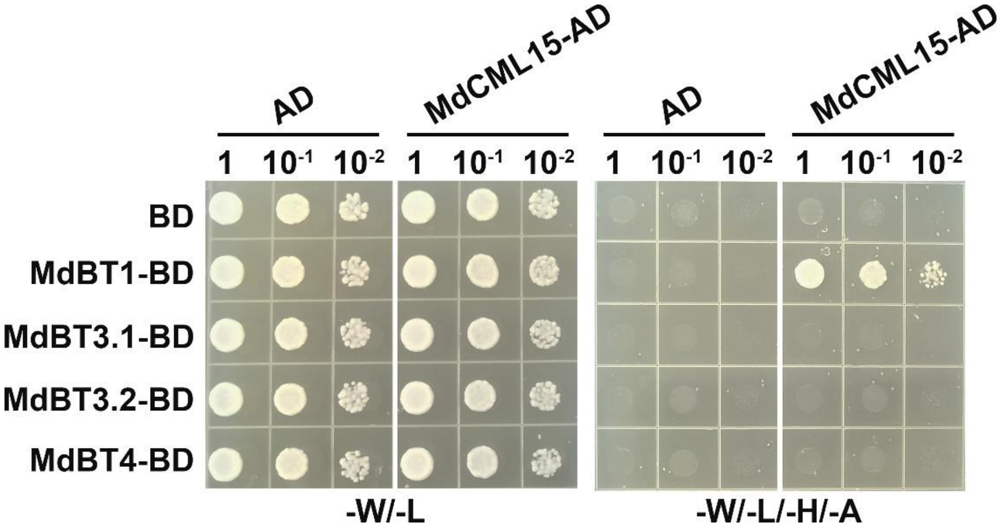

Figure S2 Y2H assay of the interaction between MdCML15 and MdBTs.

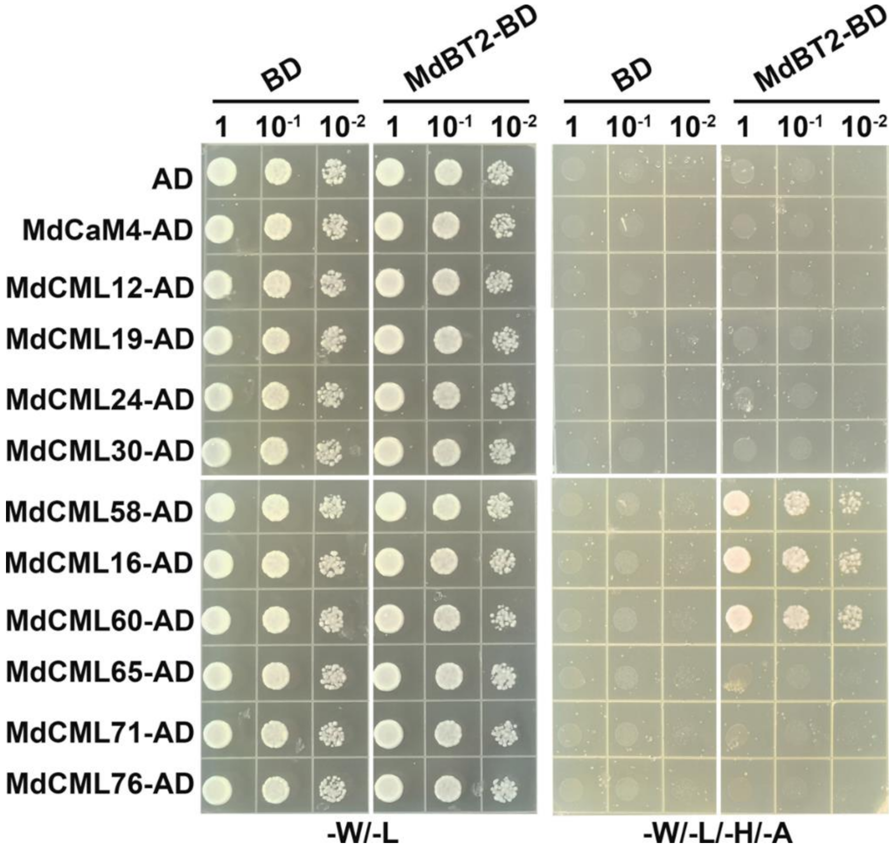

Figure S3 Y2H assay of the interaction between MdBT2 and MdCMLs.

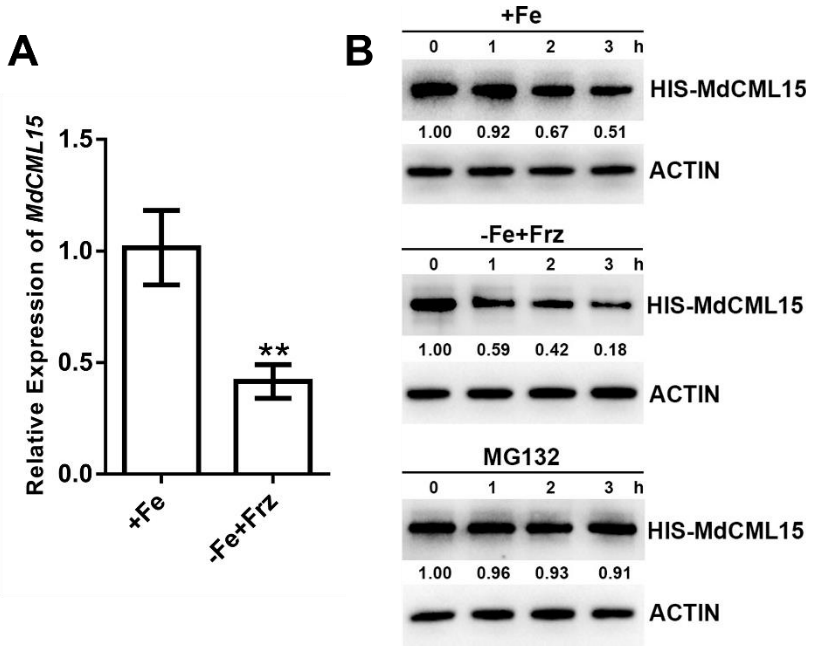

Figure S4 The transcriptional and posttranslational levels of MdCML15 in response to Fe deficiency.

(A) RT-qPCR analysis of *MdCML15* in response to Fe deficiency. 30-d-old apple plantlets (with roots) were treated with +Fe or -Fe+Frz for 1 d. The error bars indicate the SDs of three independent biological replicates. The asterisks indicate significant differences (**P < 0.01) based on Student's *t* test. **(B)** Cell-free degradation assays of MdCML15 in response to Fe deficiency. 30-d-old apple plantlets (with roots) were treated with +Fe or -Fe+Frz for 2 d. The total protein extracts from these plantlet roots and recombinant HIS-MdCML15 proteins were incubated together for the indicated times. Anti-HIS antibody was used for immunoblotting. ACTIN was used as loading control. The protein levels at 0 h were set to 1.00.

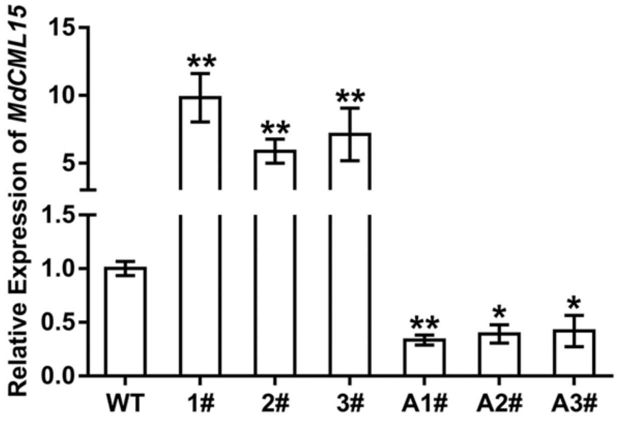

Figure S5 RT-qPCR analysis of *MdCML15* in *35S::MdCML15* and *35S::asMdCML15* transgenic apple plantlets.

Error bars indicate SD of three biological replicates. *, P < 0.05; **, P < 0.01.

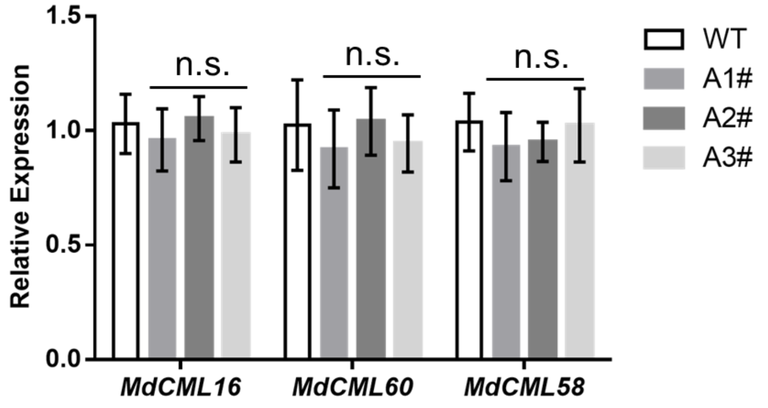

Figure S6 RT-qPCR analysis of homologous genes of *MdCML15* in *35S::asMdCML15* transgenic apple plantlets.

Error bars indicate SD of three biological replicates. n.s., P > 0.05.

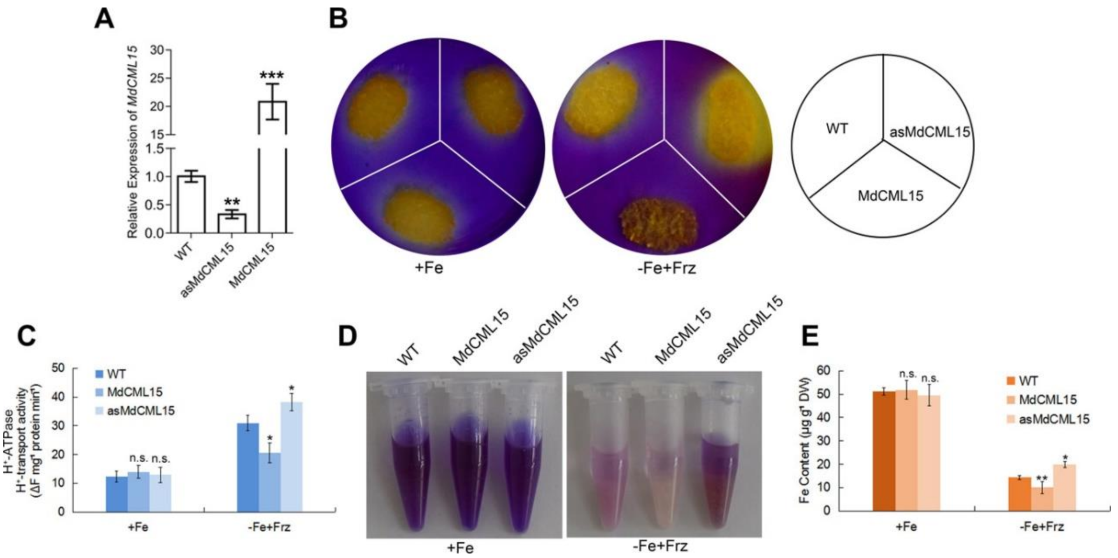

Figure S7 MdCML15 negatively regulates PM H^+-ATPase activity and Fe deficiency response in apple calli.

(A) RT-qPCR analysis of *MdCML15* in WT, *35S::MdCML15* and *35S::asMdCML15* calli. **(B)** Acidification detection of WT, *35S::MdCML15* and *35S::asMdCML15* calli grown on +Fe or -Fe+Frz media with bromocresol purple. **(C)** The PM H^+-ATPase activity of WT, *35S::MdCML15* and *35S::asMdCML15* calli grown on +Fe or -Fe+Frz media. **(D)** Visualization of ferrous in WT, *35S::MdCML15* and *35S::asMdCML15* calli under +Fe or -Fe+Frz condition for 15 d by ferrozine. **(E)** The Fe contents of WT, *35S::MdCML15* and *35S::asMdCML15* calli grown on +Fe or -Fe+Frz media. In (A), (C) and (E), error bars indicate SD of three biological replicates. n.s., $P > 0.05$; *, $P < 0.05$; **, $P < 0.01$; ***, $P < 0.001$.

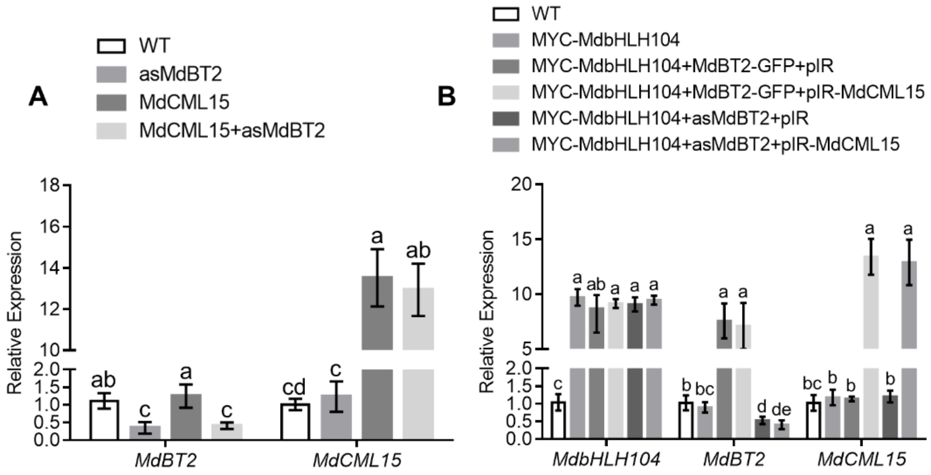

Figure S8 RT-qPCR analysis of *MdCML15*, *MdBT2* and *MdbHLH104* in related transgenic calli.

(A) The expression level of *MdCML15* and *MdBT2* in *35S::MdCML15*, *35S::asMdBT2* and *35S::MdCML15 + 35S::asMdBT2* calli. **(B)** RT-qPCR analysis of *MdbHLH104*, *MdBT2* and *MdCML15* in the indicated calli. *MdCML15* was transiently over-expressed in *35S::MYC-MdbHLH104 + 35S::MdBT2-GFP* and *35S::MYC-MdbHLH104 + 35S::asMdBT2* calli. Different letters represent significant difference at P < 0.05 (one-way ANOVA, Tukey's test), and error bars indicate SD of three biological replicates.

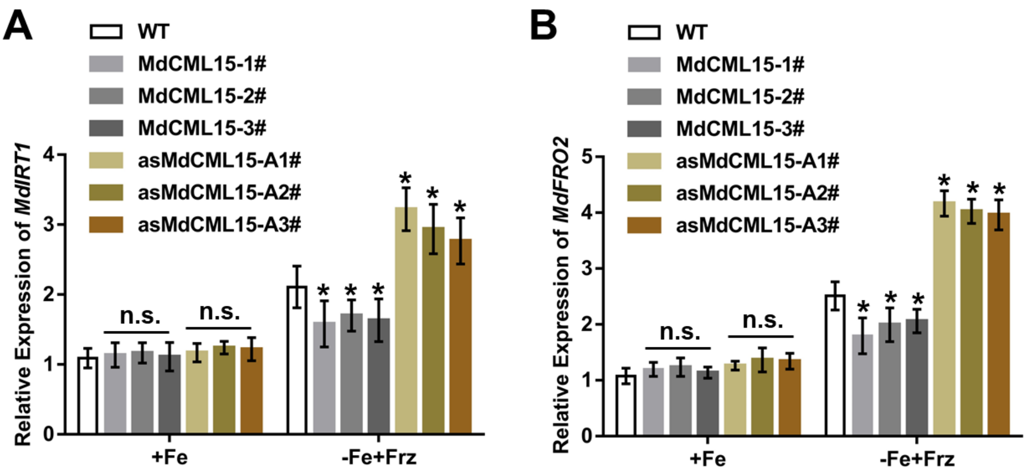

Figure S9 RT-qPCR analysis of *MdIRT1* and *MdFRO2* in the roots of WT, *35S::MdCML15* and *35S::asMdCML15* transgenic plants.

Error bars indicate SD of three biological replicates. n.s., P > 0.05; *, P < 0.05.

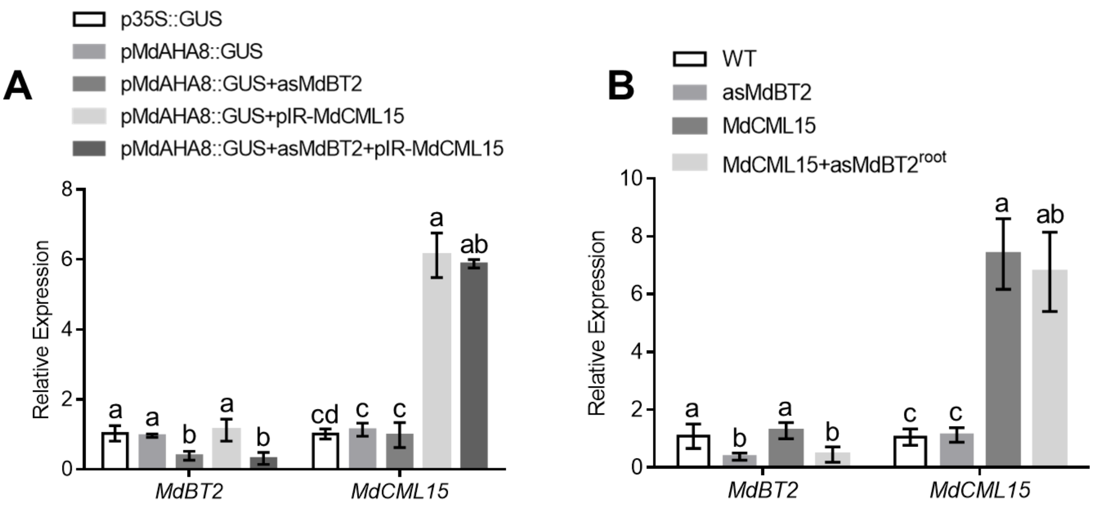

Figure S10 RT-qPCR analysis of *MdCML15* and *MdBT2* in transgenic calli and plantlets.

(A) The expression level of *MdBT2* and *MdCML15* in the indicated calli. *MdCML15* was transiently over-expressed in *pMdAHA8::GUS* and *pMdAHA8::GUS* + *35S::asMdBT2* calli. **(B)** RT-qPCR analysis of *MdBT2* and *MdCML15* in the indicated plantlets root. *MdBT2* was suppressed in WT and *35S::MdCML15* transgenic plantlet roots. Different letters represent significant difference at $P < 0.05$ (one-way ANOVA, Tukey's test), and error bars indicate SD of three biological replicates.

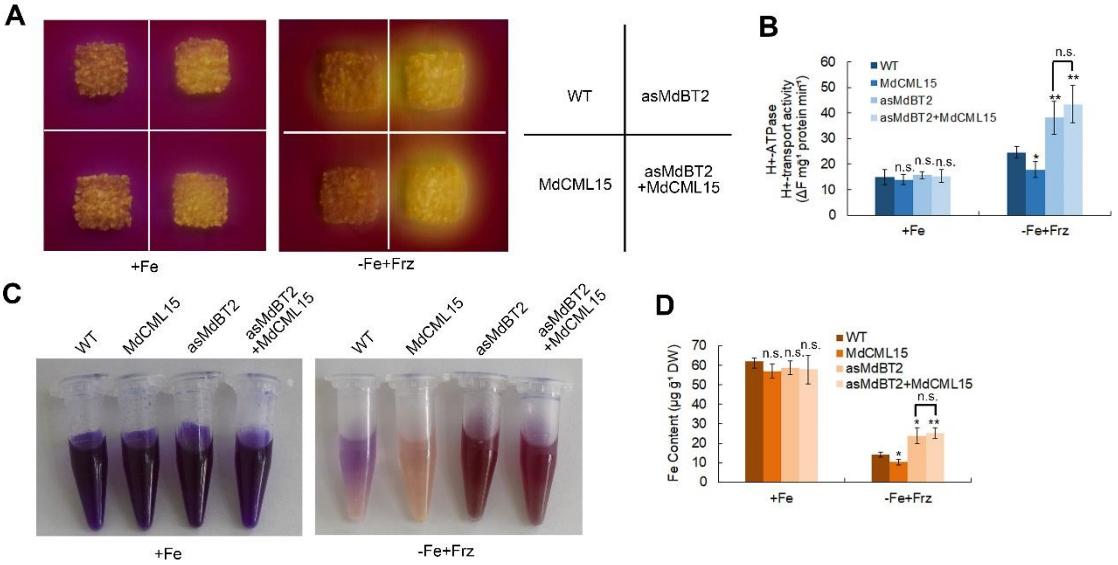

Figure S11 MdBT2 is necessary for MdCML15-mediated Fe deficiency response in apple calli.

The calli (WT, *35S::asMdBT2*, *35S::MdCML15* and *35S::MdCML15* + *35S::asMdBT2*) were grown on +Fe or –Fe+Frz media for 20 d. **(A)** Acidification analysis of these calli with bromocresol purple for 1 d. **(B)** The PM H^+-ATPase activity of these calli. **(C)** Visualization of ferrous in these calli by ferrozine. **(D)** The Fe contents of these calli. In (B) and (D), error bars indicate SD of three biological replicates. n.s., $P > 0.05$; *, $P < 0.05$; **, $P < 0.01$.

Table S1 List of primers used in this study.

Name	Primers sequences
MdCML15	(RT)-F: CAGACAGCCCAACCCTACAG<br>(RT)-R: CCTCCGCCTTGATTCTTGT
MdBT2	(RT)-F: TCGAACTCCACATCATGCAATT<br>(RT)-R: CCATTCACCCTCCTCTTACAC
MdCML16	(RT)-F: AGGTCAAGGTCAACCCTAC<br>(RT)-R: CTCCTCCACCTTGATTC
MdCML60	(RT)-F: TTGCGTAGGTTGTGTGTTGC<br>(RT)-R: CCAGACTCTGAAGCCTCGTT
MdCML58	(RT)-F: TAAGGAGAAGGACAAAGCGTA<br>(RT)-R: CAATGAGGAATGGCAGGA
MdAHA8	(RT)-F: TCGGTGTGAATGTGAAGATGAT<br>(RT)-R: TCAGGAAATACTCCAGCAAATC
MdFRO2	(RT)-F: AATCAGACGAAAGGCTAACTCAA<br>(RT)-R: GAAGATAGATACAACCCAACACC
MdIRT1	(RT)-F: TTTCTGGCTTTGTGGCTATGTT<br>(RT)-R: GGCTGGAGTTTCACCATTATCT
MdCML15 (pENTR)	F: CACCATGTCAGGCTACCCACACC<br>R: CGCAATGAGGAATGGCAGGAC
MdCML15 (pXSN)	F: AATGTCAGGCTACCCACACC<br>R: CTACGCAATGAGGAATGGCAGGA
asMdCML15 (pXSN)	F: ACAGACAGCCCAACCCTACAG<br>R: GGTGAAGGTGTACATGAGAAGA
MdCML15 (pIR)	F: GTCGACATGTCAGGCTACC<br>R: TCTAGACGCAATGAGGAATG
MdCML15 (pGAD424)	F: GAATTCATGTCAGGCTACCCACACCCCT<br>R: GGATCCCTACGCAATGAGGAATGGCAG
MdCML15 (pGEX-4T-1)	F: GGATCCATGTCAGGCTACCCACACC<br>R: GTCGACCGCAATGAGGAATGGCAG
MdBT2 (pENTR)	F: CACCATGGAAGCTAATCCGACC<br>R: CGACTAATTAATCACAATCTG
MdBT2 (pRI-GFP)	F: GGTACCATGGAAGCTAATCCGACC<br>R: GAATTCCAATCTGAAGCTTCT
asMdBT2 (pRI)	F: GAATTCTCGAACTCCACATCATGCAATT<br>R: GTCGACCCATTCACCCTCCTCTTACAC
asMdBT2 (pK7GWIWG2)	F: CACCTCGAACTCCACATCATGCAATT<br>R: CCATTCACCCTCCTCTTACAC
MdBT2 (pGBT9)	F: GAATTCATGGAAGCTAATCCGACCGC<br>R: CTGCAGTCACAATCTGAAGCTTCT
MdBT1 (pGBT9)	F: GAATTCATGGAAGCTTCTCCGACC<br>R: CTGCAG TCACAATCTGAAGCTTCT
MdBT3.1 (pGBT9)	F: GAATTCATGGCTTCATCTACTCCG

	R: CGTCGATCATGATAAACGCGAGTG
MdBT3.2 (pGBT9)	F: GAATTCATGGCTTCACCTACTCTTG<br>R: CGTCGATCATGAAAAACGTGAGCG
MdBT4 (pGBT9)	F: GAATTCATGTGTAAGGTGAAAAAC<br>R: CGTCGATCACTGCCACAAAGTGCTG
MdBT2 (pET32a)	F: GAATTCATGGAAGCTAATCCGACCGC<br>R: CTCGAGTCACAATCTGAAGCTTCT
MdbHLH104 (pET32a)	F: GGATCCATGGGGGAATGGATAGAGTAT<br>R: GTCGACAGCAGCAGGGGGCCTAAG
MdCML15 EF-Hand (pGAD424)	F: GAATTCATGTCAGGCTACCCACACCCT<br>R: GGATCCGCTCCCGTAACCATGAGCAG
MdCML15△EF-Hand (pGAD424)	F: GAATTCCCGTTTGCGGCTTTGTTG<br>R: GGATCCCTACGCAATGAGGAATGGCAG
MdBT2 BTB (pGBT9)	F: GAATTCATGGAAGCTAATCCGACC<br>R: CTGCAGCTTCTTCCTCGATTCAATT
MdBT2 TAZ (pGBT9)	F: GAATTCGAAAACGTGGTGGACGTGC<br>R: CTGCAGGCAAAGAGGGACTCTGC
MdBT2 CaMBD (pGBT9)	F: GAATTCGCAGAGTCCCTCTTTGC<br>R: CTGCAGCAATCTGAAGCTTCTAAT
MdBT2 BTB+TAZ (pGBT9)	F: GAATTCATGGAAGCTAATCCGACC<br>R: CTGCAGGCAAAGAGGGACTCTGC
MdBT2 TAZ+CaMBD (pGBT9)	F: GAATTCGAAAACGTGGTGGACGTGC<br>R: CTGCAGCAATCTGAAGCTTCTAAT
